# Machine learning-based prediction model for bronchoalveolar lavage efficacy in severe pneumonia

**DOI:** 10.3389/fmed.2025.1710355

**Published:** 2026-01-07

**Authors:** Ning Zhang, Lin Cui, Xiaojun Zhang

**Affiliations:** 1 Department of Emergency, Qilu Hospital (Qingdao), Cheeloo College of Medicine, Shandong University, Qingdao, China; 2 Department of Pathology, Qingdao Municipal Hospital, Qingdao, China

**Keywords:** bronchoalveolar lavage, community-acquired severe pneumonia, efficacy, machine learning, predictive model

## Abstract

**Objective:**

The objective of the study was to construct and validate a predictive model for the clinical efficacy of bronchoalveolar lavage (BAL) with fiberoptic bronchoscopy in patients with community-acquired severe pneumonia based on inflammatory response indicators and blood gas analysis results.

**Methods:**

A total of 206 patients with severe pneumonia who underwent BAL treatment in our hospital from November 2020 to November 2024 were enrolled and randomly divided into a training set (*n* = 144) and a validation set (*n* = 62) in a 7:3 ratio. In the training set, efficacy-related indicators were screened using univariate analysis. After variable selection via a least absolute shrinkage and selection operator (LASSO) regression analysis, independent factors influencing poor efficacy were determined using multivariate logistic regression analysis. Random forest (RF), K-nearest neighbor algorithm (KNN), and gradient boosting (GB) models were constructed using Python. The performance of the models was evaluated by the area under (AUC) the receiver operating characteristic curve (ROC), and the optimal model was selected.

**Results:**

There were 32 cases (22.22%) and 13 cases (20.97%) with poor clinical efficacy in the training set and the validation set, respectively. In the training set, the multivariate logistic regression analysis showed that age ≥ 60 years, comorbid chronic obstructive pulmonary disease (COPD), procalcitonin (PCT) ≥ 2 ng/mL, C-reactive protein (CRP) ≥ 100 mg/L, partial pressure of arterial oxygen (PaO₂) < 60 mmHg, and partial pressure of arterial carbon dioxide (PaCO₂) ≥ 50 mmHg were independent risk factors for poor efficacy (all *p* < 0.05). The AUC values of the RF model (0.799 in the training set and 0.778 in the validation set) were significantly higher than those of the KNN (0.759, 0.721) and the GB (0.766, 0.738) models, making it the optimal predictive model.

**Conclusion:**

An RF-based predictive model was successfully developed and validated to predict the efficacy of BAL treatment in patients with severe pneumonia. This model effectively identifies and quantifies key factors influencing BAL treatment outcomes in severe pneumonia, providing basic data support for subsequent hypothesis testing and clinical efficacy prediction research. However, as this is a single-center, hypothesis-generating study, the application of the model in individualized treatment planning still requires multicenter external validation and further analysis including a non-BAL control group.

## Introduction

Severe pneumonia is an acute and life-threatening respiratory condition associated with high mortality, posing significant challenges in clinical management (1). Bronchoalveolar lavage (BAL) with fiberoptic bronchoscopy is primarily a diagnostic procedure but can also serve as an adjunctive therapeutic intervention in patients with severe pneumonia who have significant secretion retention, aiding airway clearance and improving ventilation (2). However, the clinical efficacy of BAL treatment varies significantly among patients, and poor efficacy not only worsens the clinical prognosis but also complicates treatment decision-making. Therefore, accurately predicting the clinical efficacy of BAL in the treatment of severe pneumonia is crucial for optimizing treatment plans and improving patient outcomes (3). Currently, there are relatively few studies on predicting the clinical efficacy of BAL in patients with severe pneumonia, and effective prediction models are lacking. As a visual prediction tool, the random forest (RF) model integrates multiple influencing factors and can intuitively predict the probability of an individual experiencing a specific event. It has shown good application prospects in the prognosis evaluation of various diseases (4). This study aims to construct an RF model for predicting the clinical efficacy of BAL in patients with severe pneumonia using machine learning techniques. By thoroughly analyzing relevant risk factors, it provides a quantitative basis for clinicians to evaluate the efficacy of patients before treatment, aids in formulating more accurate and personalized treatment plans, and ultimately improves the overall treatment management of severe pneumonia and patient prognosis.

## Materials and methods

### Study population

A total of 206 patients diagnosed with community-acquired severe pneumonia who received BAL treatment between November 2020 and November 2024 were enrolled. The inclusion criteria were as follows: patients who met the diagnostic criteria for severe pneumonia (5), patients who were aged ≥18 years, patients who received BAL treatment within 48 h of diagnosis, and patients or their families who signed the informed consent form. The exclusion criteria were as follows: patients who had severe dysfunction or failure of important organs such as the heart, liver, and kidneys and were unable to tolerate BAL treatment; patients who had contraindications for bronchoscopy, such as severe coagulation dysfunction and severe cardiopulmonary insufficiency; patients who used immunosuppressants or glucocorticoids within the past month; and patients who had mental diseases and were unable to cooperate with the study. The patients were randomly divided into a training set (*n* = 144) and a validation set (*n* = 62) in a ratio of 7:3 using the random number table method.

### Data collection

A standardized data extraction process was applied in the hospital. Trained research nurses and physicians collaborated to extract data from electronic medical records and case report forms, with a double-verification procedure—one researcher first extracted the data, and another independently reviewed and cross-checked it, with discrepancies resolved via discussion with the research team leader based on original medical records. General information of the patients was recorded, including age, body mass index (BMI), gender, comorbid underlying diseases (such as hypertension, diabetes, and chronic obstructive pulmonary disease [COPD]), and clinical characteristics (such as Acute Physiology and Chronic Health Evaluation II [APACHE II] score). Peripheral venous blood was collected from the patients before BAL treatment to detect inflammatory response indicators, such as procalcitonin (PCT), C-reactive protein (CRP), and white blood cell (WBC) count. At the same time, arterial blood was collected for blood gas analysis to detect partial pressure of arterial oxygen (PaO₂), partial pressure of arterial carbon dioxide (PaCO₂), arterial oxygen saturation (SaO₂), and the results of BAL pathogen detection.

### Treatment method

All patients received BAL treatment. The operation method was as follows: the patients were placed in the supine position, given routine oxygen inhalation, and their vital signs were monitored. A fiberoptic bronchoscope was inserted through the nasal cavity or oral cavity. After reaching the lesion site, 37 °C sterile normal saline was used for lavage. The lavage volume each time was 20–50 mL, and the total volume did not exceed 200 mL. After the lavage fluid was recovered, it was sent for bacterial culture, fungal culture, and drug sensitivity tests. Drug susceptibility testing was conducted via the broth microdilution method following the Clinical and Laboratory Standards Institute (CLSI) guidelines: pure bacterial colonies from lavage fluid culture were prepared into suspensions meeting the CLSI McFarland turbidity standard, inoculated into 96-well microtiter plates with gradient antimicrobial concentrations, incubated at 35 °C for 16–20 h aerobically, and the minimum inhibitory concentration (MIC) was determined. The results were interpreted as susceptible, intermediate, or resistant based on CLSI MIC breakpoints. After the operation, the patients were given comprehensive treatment such as anti-infection, expectoration, and support. Expectorant therapy involved nebulized administration of 15 mg of ambroxol hydrochloride mixed with 5 mL of normal saline, delivered twice daily for 10–15 min each session to dilute airway secretions. Supportive care included monitoring serum electrolytes and blood glucose to maintain fluid–electrolyte balance; prioritizing enteral nutrition (initially 20–30 mL/h, gradually increasing to target dose based on tolerance) for patients with intact gastrointestinal function and parenteral nutrition for those with dysfunction; recording vital signs (heart rate, blood pressure, respiratory rate, temperature, and SaO₂) every 1–2 h; and adjusting oxygen concentration/delivery mode to maintain SaO₂ ≥ 90%.

### Outcome definition

Referring to the “Technical Guidelines for Antibacterial Drug Clinical Trials” of the US Food and Drug Administration (FDA) and the “Guidelines for the Diagnosis and Treatment of Community – Acquired Pneumonia in Chinese Adults,” patients were evaluated after 7 days of treatment based on symptoms, signs, laboratory tests, and imaging results. Cure was defined as the disappearance of symptoms and signs, normalization of laboratory test indicators, and complete absorption of pulmonary inflammation on chest imaging. Marked effect: Symptoms and signs were significantly relieved (e.g., body temperature returned to normal and respiratory rate ≤ 20 breaths/min), inflammatory indicators (CRP and PCT) decreased by ≥ 50% compared with baseline, and chest imaging showed ≥ 50% absorption of pulmonary inflammation. Effective: Symptoms and signs were improved (e.g., body temperature decreased by ≥ 1 °C compared with baseline and respiratory rate decreased by ≥ 5 breaths/min compared with baseline), inflammatory indicators decreased by 20–49% compared with baseline, and chest imaging showed < 50% absorption of pulmonary inflammation (6). Ineffective: Symptoms and signs did not improve or worsened, laboratory test indicators did not improve or deteriorated, and chest imaging showed no absorption or progression of pulmonary inflammation. Cure, marked effect, and effective outcomes were classified as good clinical efficacy, whereas ineffective outcomes were classified as poor clinical efficacy. This grouping aimed to identify risk factors associated with poor clinical outcomes, thereby providing a basis for early identification of high-risk patients and the development of personalized intervention strategies.

### Sample size calculation

The main outcome indicator was clinical efficacy, a binary variable classified as “good efficacy” and “poor efficacy.” Based on the pre-experiment and previous literature, the proportion of poor efficacy in the training set was approximately 22.2% (32/144) (7). Machine learning algorithms, including logistic regression, RF, K-nearest neighbor algorithm (KNN), and gradient boosting (GB), were used for model development and validation. The sample size of the machine learning prediction model was determined to meet the events per variable (EPV) criterion. The training set had 32 poor efficacy events. Since EPV being ≥5 is an acceptable minimum for exploratory studies with limited samples, we calculated the maximum variable number as 32/5 = 6.4 (up to 6 variables in the final model) (8). To mitigate overfitting, we further used LASSO regression analysis for variable selection and the RF algorithm for model construction. The pwr package (R language) was used for verification, assuming *α* = 0.05, 1−*β* = 80%, the effect size OR = 2.5 (moderate effect), the number of prediction variables was 5, and the total number of required events was 50. The actual number of events in this study was 46 (206 × 22.3%), which was close to the minimum requirement. It could be considered that the sample size basically met the needs of exploratory prediction model research. Considering a 10–15% missing value or loss to follow-up rate, the actual sample size had a slight surplus, supporting complete case analysis.

### Statistical analysis

SPSS 26.0, Python 3.8.5, and R 4.2.3 software were used for statistical analysis. Measurement data that conformed to the normal distribution were expressed as x̄ ± s, and the *t*-test was used for comparison between the groups. Data that did not conform to the normal distribution were expressed as median (interquartile range), and the Mann–Whitney U-test was used. Count data were expressed as the number of cases (percentage), and the χ^2^ test was used for comparison between the groups. In the training set, a univariate analysis was first performed to screen out indicators. After variable selection by a least absolute shrinkage and selection operator (LASSO) regression analysis, a multivariate logistic regression analysis was used to determine independent influencing factors, and their odds ratios (OR) and 95% confidence intervals (CI) were calculated. Variance inflation factors (VIF) were calculated to exclude multicollinearity (VIF threshold <10). Based on the selected predictive indicators, the RF model, KNN, and GB models were constructed. The receiver operating characteristic (ROC) curve was plotted, and the area under the curve (AUC) value was calculated. A *p*-value of < 0.05 was considered statistically significant.

## Results

### Comparison of general information and clinical efficacy between the training set and the validation set

In the training set (*n* = 144), 32 patients (22.22%) had poor clinical efficacy, and in the validation set (*n* = 62), 13 patients (20.97%) had poor clinical efficacy. There were no statistically significant differences between the two groups of patients in terms of age, gender, underlying diseases, clinical characteristics, inflammatory response indicators, blood gas analysis indicators, and the incidence of poor clinical efficacy (all *p* > 0.05), indicating comparability (Table 1).

**Table 1:** Comparison of general information of patients in the training set and the validation set.

Indicators		Training set (*n* = 144)	Validation set (*n* = 62)	χ^2^/*t*	*P*
Age (years)	≥60	56 (38.89)	22 (35.48)	0.213	0.644
	<60	88 (61.11)	40 (64.52)		
BMI	≥25 kg/m^2^	48 (33.33)	18 (29.03)	0.368	0.544
	<25 kg/m^2^	96 (66.67)	44 (70.97)		
Gender	Male	78 (54.17)	30 (48.39)	0.580	0.446
	Female	66 (45.83)	32 (51.61)		
Comorbid diabetes	Yes	24 (16.67)	10 (16.13)	0.009	0.924
	No	120 (83.33)	52 (83.87)		
Comorbid hypertension	Yes	36 (25.00)	14 (22.58)	0.138	0.710
	No	108 (75.00)	48 (77.42)		
Comorbid COPD	Yes	18 (12.50)	7 (11.29)	0.059	0.807
	No	126 (87.50)	55 (88.71)		
APACHE II score	≥15 points	42 (29.17)	16 (25.81)	0.241	0.622
	<15 points	102 (70.83)	46 (74.19)		
PCT	≥2 ng/mL	30 (20.83)	12 (19.35)	0.058	0.809
	<2 ng/mL	114 (79.17)	50 (80.63)		
CRP	≥100 mg/L	60 (41.67)	24 (38.71)	0.156	0.692
	<100 mg/L	84 (58.33)	38 (61.29)		
WBC	≥12 × 10^9^/L	48 (33.33)	18 (29.03)	0.368	0.544
	<12 × 10^9^/L	96 (66.67)	44 (70.97)		
PaO₂	≥60 mmHg	108 (75.00)	46 (74.19)	0.014	0.902
	<60 mmHg	36 (25.00)	16 (25.81)		
PaCO₂	≥50 mmHg	37 (25.69)	14 (22.58)	0.225	0.634
	<50 mmHg	107 (74.31)	48 (77.42)		
SaO₂	≥90%	131 (90.97)	56 (90.32)	0.021	0.882
	<90%	13 (9.03)	6 (9.68)		

BMI, body mass index; COPD, chronic obstructive pulmonary disease; PCT, procalcitonin; CRP, C-reactive protein; WBC, white blood cell count; PaO₂, partial pressure of arterial oxygen; PaCO₂, partial pressure of arterial carbon dioxide; SaO₂, arterial oxygen saturation; APACHE II, Acute Physiology and Chronic Health Evaluation II. χ^2^, chi-squared test for categorical variables; *t*, *t*-test for normally distributed continuous variables. Data presentation: categorical variables as *n* (%), normally distributed continuous variables as mean ± SD. A *P*-value of < 0.05 indicates a significant difference.

### Univariate analysis of influencing factors for clinical efficacy in the training set

In the training set, the results of the univariate analysis showed that there were statistically significant differences in age, COPD, PCT, CRP, PaO₂, and PaCO₂ (all *p* < 0.05) (Table 2).

**Table 2:** Univariate analysis of influencing factors for clinical efficacy in the training set.

Indicators		Poor efficacy (*n* = 32)	Good efficacy (*n* = 112)	χ^2^/*t*	*P*
Age (years)	≥60	19 (59.38)	37 (33.04)	7.265	0.007
	<60	13 (40.62)	75 (66.96)		
BMI	≥25 kg/m^2^	13 (40.62)	35 (31.25)	0.984	0.321
	<25 kg/m^2^	19 (59.38)	77 (63.11)		
Gender	Male	18 (56.25)	60 (53.57)	0.071	0.788
	Female	14 (43.75)	52 (46.43)		
Comorbid diabetes	Yes	6 (18.75)	18 (16.07)	0.128	0.719
	No	26 (81.25)	94 (83.93)		
Comorbid hypertension	Yes	9 (28.13)	27 (24.11)	0.214	0.643
	No	23 (71.87)	85 (75.89)		
Comorbid COPD	Yes	8 (25.00)	10 (8.93)	4.500	0.033
	No	24 (75.00)	102 (91.07)		
APACHE II score	≥15 points	12 (37.50)	30 (26.79)	1.383	0.239
	<15 points	20 (62.50)	82 (73.21)		
PCT	≥2 ng/mL	11 (34.38)	19 (16.96)	4.574	0.032
	<2 ng/mL	21 (65.62)	93 (83.04)		
CRP	≥100 mg/L	19 (59.38)	41 (36.61)	5.308	0.021
	<100 mg/L	13 (40.62)	71 (63.39)		
WBC	≥12 × 10^9^/L	12 (37.50)	36 (32.14)	0.321	0.570
	<12 × 10^9^/L	20 (62.50)	76 (67.86)		
PaO₂	≥60 mmHg	18 (56.25)	90 (80.36)	7.143	0.005
	<60 mmHg	14 (43.75)	22 (19.64)		
PaCO₂	≥50 mmHg	14 (43.75)	23 (20.54)	7.025	0.008
	<50 mmHg	18 (56.25)	89 (79.46)		
SaO₂	≥90%	27 (84.38)	104 (92.86)	1.269	0.259
	<90%	5 (15.62)	8 (7.14)		

BMI, body mass index; COPD, chronic obstructive pulmonary disease; PCT, procalcitonin; CRP, C-reactive protein; WBC, white blood cell count; PaO₂, partial pressure of arterial oxygen; PaCO₂, partial pressure of arterial carbon dioxide; SaO₂, arterial oxygen saturation; APACHE II, Acute Physiology and Chronic Health Evaluation II. χ^2^, chi-squared test for categorical variables; *t*, *t*-test for normally distributed continuous variables. Data presentation: categorical variables as *n* (%), normally distributed continuous variables as mean ± SD. A *P*-value of <0.05 indicates a significant difference.

### Multivariate logistic regression analysis

**Figure 1:**
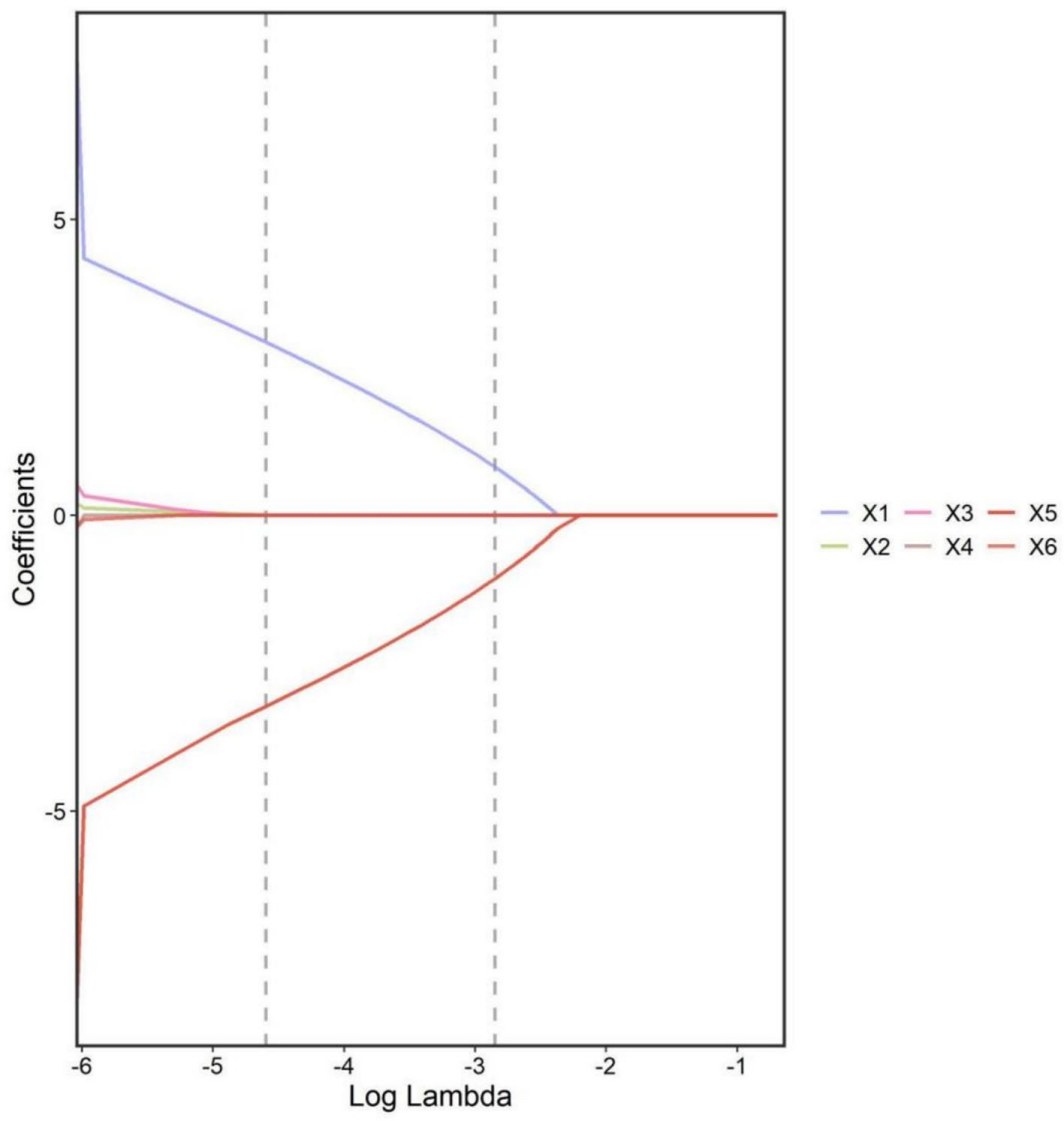
Coefficient paths for variables across log(lambda) values in the LASSO regression model. Coefficients shrink toward zero as lambda increases. Vertical dashed lines indicate the lambda.1se (right) and lambda.min (left) values. Predictors: X1: age, X2: comorbid COPD, X3: PCT, X4: CRP, X5: PaO₂, and X6: PaCO₂.

**Figure 2:**
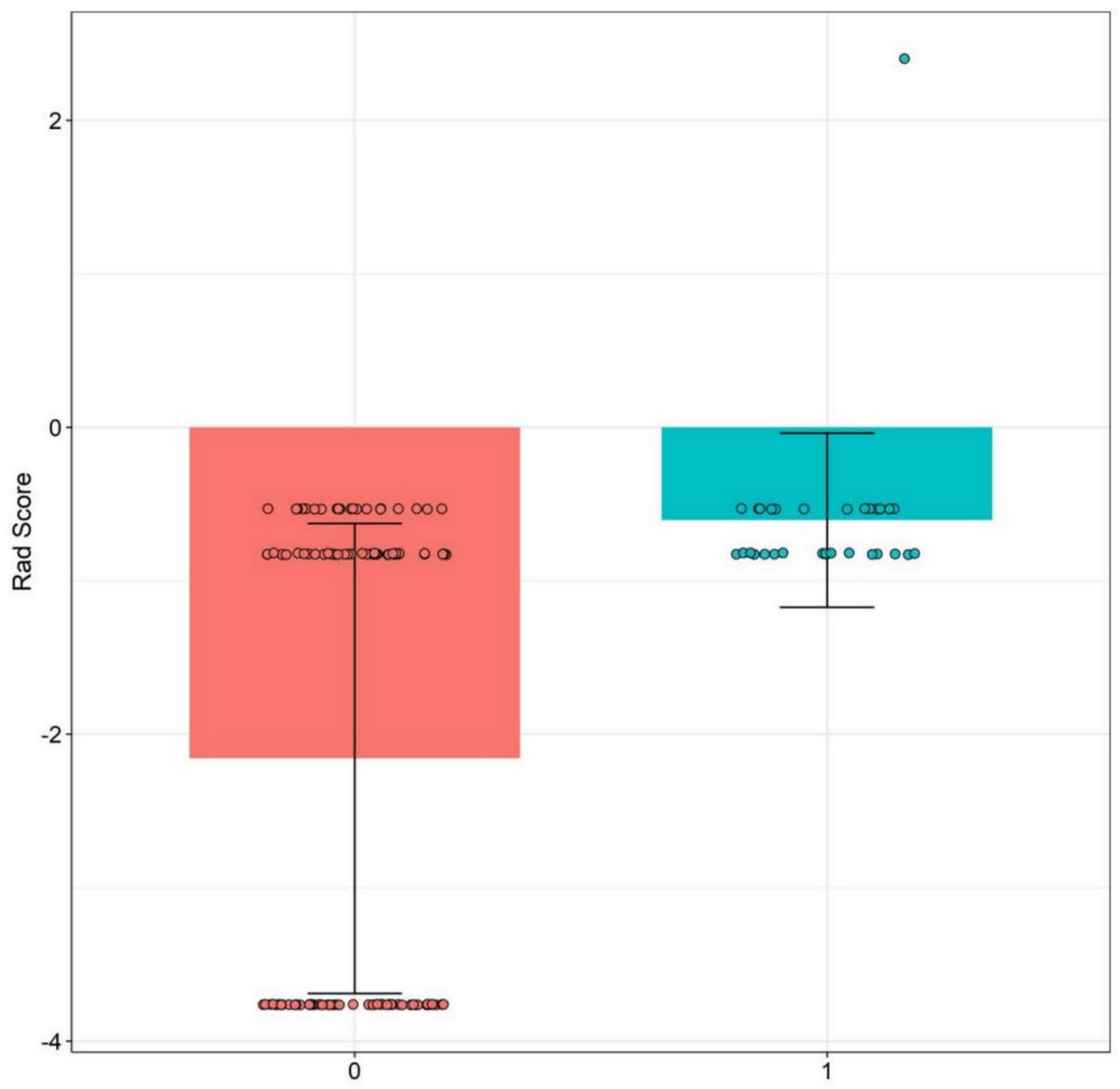
Comparison of standardized coefficients of predictors in the LASSO regression analysis. Each bar represents the standardized coefficient of a predictor: positive coefficients indicate risk factors for poor BAL efficacy, negative coefficients indicate protective factors, and bar length reflects effect magnitude. The original “Rad score” was a mislabel; the figure presents standardized coefficients of predictors (X1: age, X2: comorbid COPD, X3: PCT, X4: CRP, X5: PaO₂, and X6: PaCO₂).

Taking the treatment effect as the dependent variable (good efficacy = 0, poor efficacy = 1), the indicators with statistical significance in the univariate analysis were included in the LASSO regression analysis for variable screening (Table 3). Variables were screened using the screening criterion of lambda.1se—defined as the largest *λ* in the LASSO regression analysis where cross-validated error is within 1 standard error of the minimum cross-validated error, which balances model simplicity and predictive performance while reducing overfitting (Figures 1, 2). The appropriate predictive variables were age ≥ 60 years, comorbid COPD, PCT ≥ 2 ng/mL, CRP ≥ 100 mg/L, PaO₂ < 60 mmHg, and PaCO₂ ≥ 50 mmHg. The results of the multivariate logistic regression analysis showed that age ≥ 60 years, comorbid COPD, PCT ≥ 2 ng/mL, CRP ≥ 100 mg/L, PaO₂ < 60 mmHg, and PaCO₂ ≥ 50 mmHg were independent risk factors for poor clinical efficacy of BAL in the treatment of severe pneumonia patients (all *p* < 0.05) (Table 4). In the regression model, the tolerance of each variable was > 0.1, the VIF was < 10, and the condition index was <30. Moreover, there was no situation where the variance proportion of multiple covariates under the same eigenvalue was > 50%. Therefore, there was no collinearity among the covariates.

**Table 3:** Variable assignment methods.

Variables	Meaning	Assignment
X1	Age	0 = <60,1 = ≥60
X2	Comorbid COPD	0 = No, 1 = Yes
X3	PCT	1 = <2 ng/mL,0 = ≥2 ng/mL
X4	CRP	0 = <100 mg/L,1 = ≥100 mg/L
X5	PaO₂	0 = <60 mmHg,1 = ≥60 mmHg
X6	PaCO₂	0 = <50 mmHg,1 = ≥50 mmHg
Y	Treatment effect	Poor efficacy = 1, good efficacy = 0

COPD, chronic obstructive pulmonary disease; PCT, procalcitonin; CRP, C-reactive protein; PaO₂, partial pressure of arterial oxygen; PaCO₂, partial pressure of arterial carbon dioxide.

**Table 4:** Multivariate analysis of poor clinical efficacy in the training set.

Factor	*β*	SE	*Wald*	*P*	*OR*	95%CI
Age	1.086	0.412	6.942	0.008	2.963	1.321 ~ 6.646
Comorbid COPD	1.224	0.526	5.417	0.020	3.400	1.213 ~ 9.529
PCT	0.942	0.449	4.390	0.036	2.564	1.063 ~ 6.186
CRP	0.929	0.410	5.132	0.023	2.531	1.133 ~ 5.652
PaO₂	−1.157	0.428	7.299	0.007	0.314	0.136 ~ 0.728
PaCO₂	1.102	0.426	6.682	0.010	3.010	1.305 ~ 6.940

COPD, chronic obstructive pulmonary disease; PCT, procalcitonin; CRP, C-reactive protein; PaO₂, partial pressure of arterial oxygen; PaCO₂, partial pressure of arterial carbon dioxide; *β*, regression coefficient; SE, standard error; OR, odds ratio; CI, confidence interval.

### Predictive performance of machine learning models in the training set and validation set

**Figure 3:**
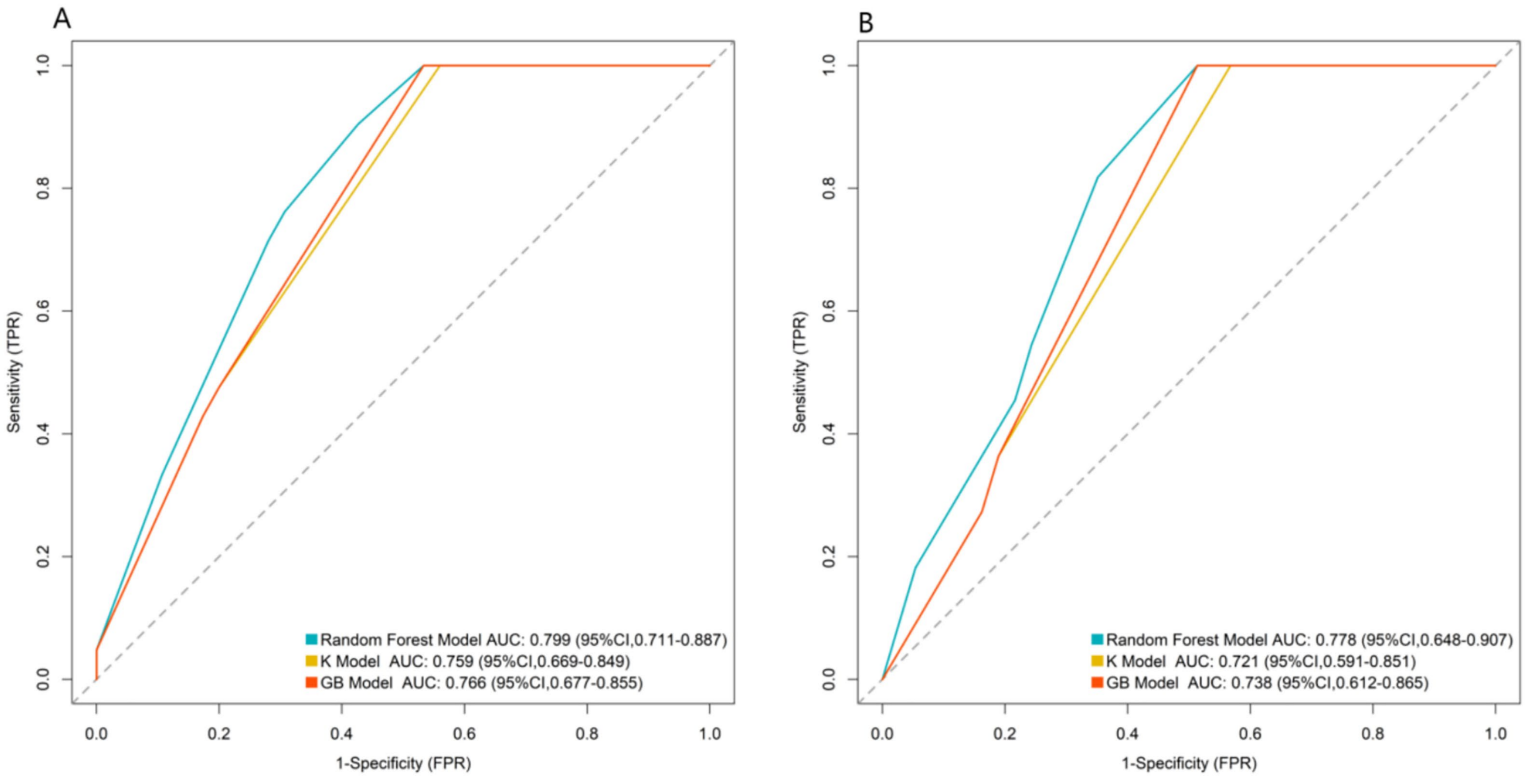
ROC curves of machine learning models (**A**: training set, **B**: validation set). Solid lines represent ROC curves of models. Dashed lines represent reference lines (sensitivity = 1-specificity, AUC = 0.5) for evaluating models’ predictive values.

The RF model, KNN, and GB model were used for prediction in the training set and the validation set. The AUC values of the three models in the training set were 0.799, 0.759, and 0.766, respectively, and the AUC values in the validation set were 0.778, 0.721, and 0.738, respectively. The model with the largest AUC value was selected as the optimal model for this study, which was the RF model (Figure 3).

### Efficacy evaluation of the machine learning prediction model

**Figure 4:**
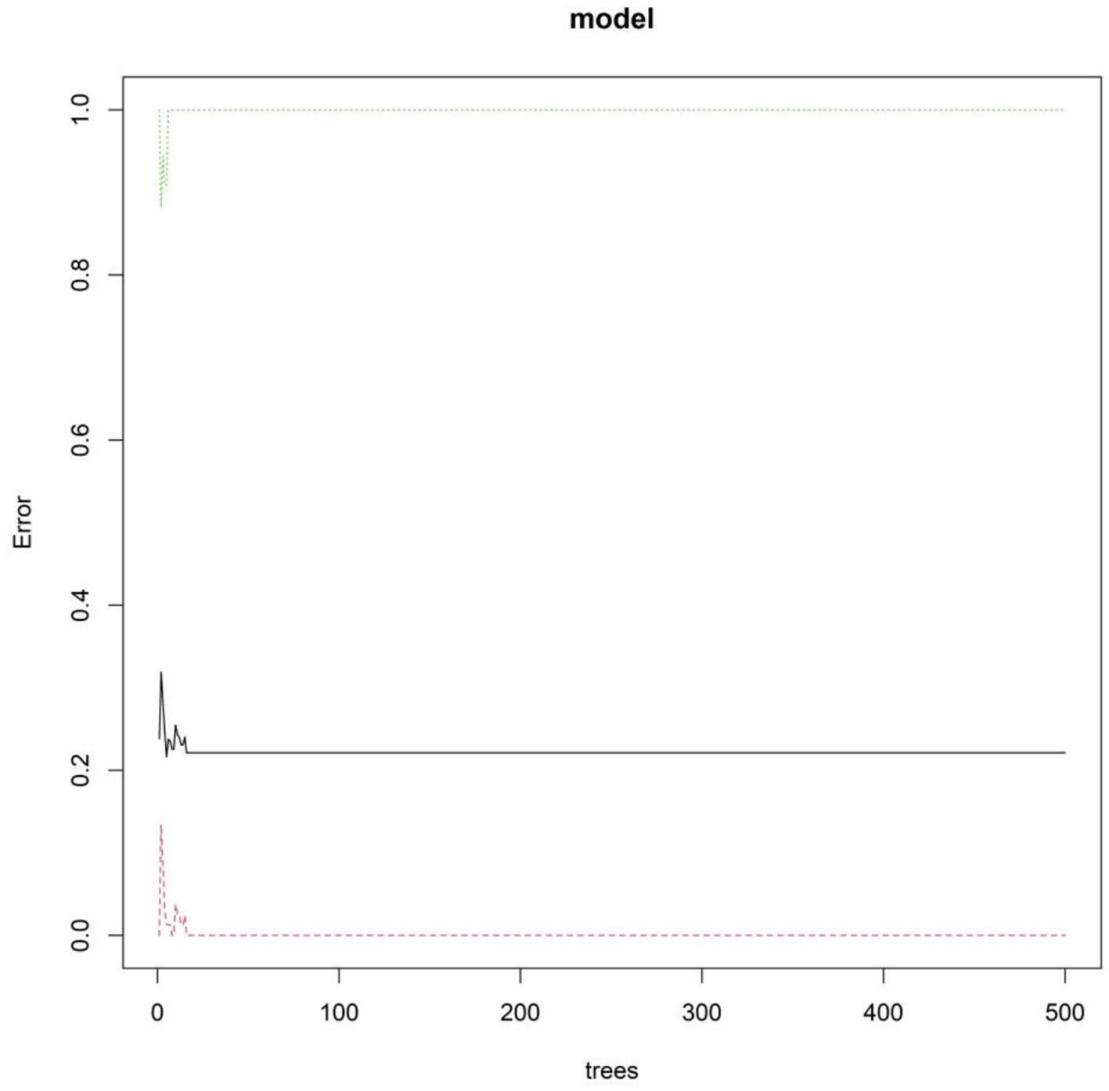
Trend of the average out-of-bag (OOB) estimation error rate with the number of decision trees. The x-axis represents the number of decision trees in the random forest (RF) model, and the y-axis represents the average OOB estimation error rate. The OOB error rate was calculated using samples not included in the bootstrap sample during each decision tree construction, reflecting the model’s generalization ability.

**Figure 5:**
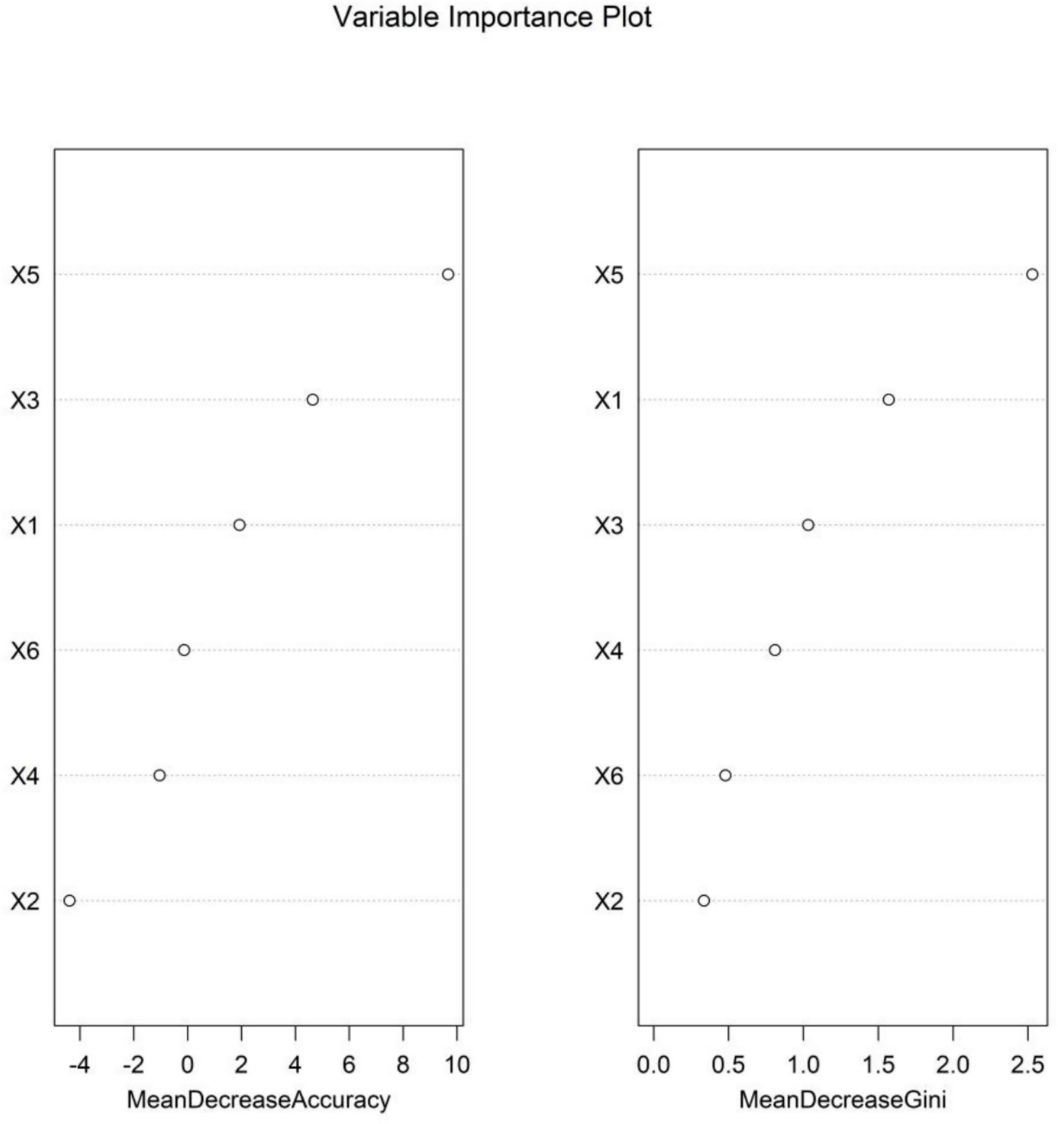
Importance ranking of the RF model. Mean decrease accuracy: decrease in model accuracy when a predictor is randomly permuted; higher values represent greater contribution to accuracy. Mean decrease Gini: average decrease in Gini impurity when a predictor splits nodes; higher values represent a stronger ability to distinguish outcomes. Predictors: X1: age, X2: comorbid COPD, X3: PCT, X4: CRP, X5: PaO₂, and X6: PaCO₂.

As the number of decision trees increased, the overall error gradually tended to be stable. This changing trend reflected the dynamic change characteristics of the predictive performance of the model during the iterative construction of decision trees. This trend could be used to assist in judging the convergence of the model. When the error curve tended to be stable, it indicated that the model complexity reached a certain level, and the optimization effect of adding new decision trees on the error was limited. This provided a basis for parameter selection to determine the optimal number of decision trees and improve the predictive efficacy of the model, helping to screen out the configuration that could balance model complexity and prediction accuracy to enhance the performance of the model in predicting the clinical efficacy of BAL in the treatment of severe pneumonia (Figure 4). Based on the RF model, the importance scores of independent influencing factors for the clinical efficacy of BAL in the treatment of severe pneumonia were calculated, and the importance ranking was as follows: PaO₂, PCT, age, PaCO₂, CRP, and comorbid COPD (Figure 5).

## Discussion

Severe pneumonia is a common and serious respiratory disease in clinical practice, and its high mortality rate has always been a severe challenge in the medical field (9). As an important means to improve the condition of patients with severe pneumonia, BAL provides strong support for precise treatment by directly clearing airway secretions and obtaining etiological specimens (10). However, there are significant differences in the treatment effects of BAL among different patients, and some patients still have poor prognoses after treatment. Therefore, exploring the factors influencing the clinical efficacy of BAL in treating severe pneumonia and constructing an effective prediction model are of great significance for optimizing treatment strategies and improving patients’ prognoses.

This study found that age ≥ 60 years old is an independent risk factor for poor clinical efficacy of BAL in patients with severe pneumonia. With an increase in age, the functions of various organs in the body gradually decline, and the immune system function also decreases accordingly. In elderly patients, the respiratory mucosa atrophies and the ciliary movement weakens, resulting in a decrease in the airway’s self-cleansing ability. They are more prone to infections, and it is difficult to clear pathogens after infection (11). At the same time, elderly patients often have multiple underlying diseases, such as cardiovascular diseases and diabetes. These diseases are intertwined, further weakening the body’s resistance and recovery ability against pneumonia. During the BAL treatment process, the physical functions of elderly patients are difficult to withstand the stress brought by the treatment, and their postoperative recovery is slow, which, in turn, affects the clinical efficacy (12). Comorbid COPD also has a negative impact on the treatment effect of BAL. Patients with COPD have persistent airway inflammation and airflow limitation, and their lung tissues are in a pathological state for a long time, resulting in an increase in airway secretions and difficulty in their discharge (13). Although BAL can clear airway secretions to a certain extent, the underlying lung lesions in COPD patients still exist, which not only affects the gas exchange function but also makes it difficult to effectively control inflammation. In addition, patients with COPD have immune dysfunction and weak defense ability against infections, making their conditions more complicated after being infected with severe pneumonia, and the treatment effect of BAL is also greatly reduced (14).

PCT ≥ 2 ng/mL and CRP ≥ 100 mg/L are key indicators reflecting the degree of the body’s inflammatory response. PCT is a glycoprotein without hormonal activity, and its content is extremely low in healthy people. When the body is severely infected by bacteria, the PCT level will rise rapidly, and the degree of its increase is positively correlated with the severity of the infection (15, 16). In this study, patients with PCT ≥ 2 ng/mL indicated that they had a severe bacterial infection in their bodies and a strong inflammatory response. In this case, BAL treatment may not be able to completely clear the pathogens in time, and the inflammation persists and further damages the lung tissue, thus affecting the clinical efficacy (17). CRP, as an acute-phase reactive protein, is rapidly synthesized by the liver and released into the bloodstream upon the onset of inflammation. A CRP level ≥ 100 mg/L indicates that the body is in a state of severe inflammation, and the massive release of inflammatory factors can damage the normal structure and function of lung tissue, hindering the repair and healing process of the lungs and making it difficult to achieve the desired therapeutic effect with BAL (18).

PaO₂ < 60 mmHg and PaCO₂ ≥ 50 mmHg reflect the severe impairment of patients’ respiratory function and the disorder of acid–base balance. PaO₂ < 60 mmHg indicates that the patient has hypoxemia, the tissues and organs do not get sufficient oxygen supply, the cell metabolism and function are inhibited, and the body’s immune function and repair ability also decline accordingly (16). While PaCO₂ ≥ 50 mmHg indicates carbon dioxide retention, which can lead to respiratory acidosis and further aggravate the pulmonary vasoconstriction, making the ventilation/perfusion ratio imbalance in the lungs more serious (19, 20). In this case, although BAL treatment can improve airway patency, its effect on correcting the damaged respiratory function and acid–base balance state is limited, thus affecting the overall treatment effect (21, 22).

The machine model constructed in this study integrates the above multiple independent risk factors. The RF model improves the prediction accuracy by integrating numerous decision trees, and the relationship between the number of decision trees and the error curve is a key indicator for evaluating whether the model training is sufficient. In this study, it was observed that, as the number of decision trees increased, the model error decreased rapidly and finally reached a stable plateau. This dynamic change process clearly shows that the model has fully learned the prediction rules from the data, and the newly added decision trees no longer make significant contributions to improving the model’s performance. This convergence trend enables the determination of the optimal model complexity parameters, effectively reducing the risk of overfitting and ensuring the generalizability of the model on unknown data. This finding indicates that the model configuration finally adopted in this study is a robust one that achieves the best balance between prediction efficiency and computational efficiency, thereby providing technical reliability for its potential application in the clinical environment.

However, this study had some limitations. First, it did not conduct external validation, primarily due to difficulties in obtaining external samples. Differences in patient population characteristics, diagnostic and treatment protocols, and detection methods among hospitals could interfere with validation results, making it challenging to accurately evaluate the effectiveness of the model in different settings. Future studies can collect more external samples through multicenter cooperation to further verify the reliability and universality of the model. Second, as a retrospective study, we did not include BAL sample microbiology indicators (e.g., pathogen type and drug resistance). Due to incomplete results (e.g., negative cultures from prior antimicrobial use and untested cases due to critical status), including such data would introduce selection bias. BAL microbiology is crucial for guiding antimicrobial therapy and evaluating efficacy, so future prospective studies should standardize microbiology testing and incorporate these indicators to enhance model comprehensiveness. Additionally, the core objective of this study is to predict the efficacy of patients receiving BAL treatment, rather than to verify the effectiveness of BAL treatment itself. Moreover, due to ethical considerations (patients with severe pneumonia who meet BAL indications need to receive timely treatment, and setting up a non-treatment control group may delay prognosis), no control group without BAL treatment was set up. Therefore, the model can only identify risk factors for poor outcomes in patients receiving BAL treatment but cannot confirm the effectiveness of BAL intervention.

Compared with traditional univariate analysis or logistic regression, the advantages of the RF algorithm are fully demonstrated in this study. This method can not only automatically handle the complex interaction effects between prediction variables but also objectively quantify the relative contribution of each variable, avoiding the subjective bias of researchers. The model constructed in this study is not a simple superposition of single factors but a multidimensional prediction system that integrates the patient’s oxygenation status, infection degree, inflammation level, age, and underlying diseases, which more comprehensively simulates the real-life scenario of clinical decision-making, thus achieving higher prediction accuracy.

In summary, age ≥ 60 years, comorbid COPD, PCT ≥ 2 ng/mL, CRP ≥ 100 mg/L, PaO₂ < 60 mmHg, and PaCO₂ ≥ 50 mmHg are independent risk factors affecting the clinical efficacy of BAL treatment. This model can effectively predict the clinical efficacy of BAL in treating patients with severe pneumonia, providing a valuable reference for clinicians to formulate individualized treatment plans. However, since this study did not conduct external validation, the universality of the model needs to be further improved. Future studies will first incorporate a control group without BAL treatment to compare and analyze the effectiveness of BAL intervention, then conduct multicenter, large-sample research for external validation, and further improve the model by incorporating additional parameters (e.g., BAL microbiology indicators, genetic factors, and treatment adherence); meanwhile, they will optimize the model threshold combined with clinical practical scenarios and gradually explore its application value in individualized treatment planning after verifying its reliability, thereby better improving the treatment level and prognosis of patients with severe pneumonia.

## Data Availability

The raw data supporting the conclusions of this article will be made available by the authors, without undue reservation.
